# 

**Published:** 1863-03

**Authors:** 


### AMERICAN MEDICAL ASSOCIATION.

OFFICE MEDICAL EXAMINER,
Chicago, February 20th. 1863.

The next regular Annual Meeting of the American Medical Association will be held in the City of Chicago, Illinois, on the first Tuesday in June, 1863. Every permanently organized State, County, and Local Medical Society is entitled to send one delegate for every ten members, and one additional delegate for a fraction of more than half of that number. Medical Colleges and Hospitals, containing over 100 beds for the sick, are entitled to two delegates; and all other permanently organized Medical Institutions are entitled to one delegate each.

The Committee earnestly desire a full attendance from all parts of the country.

By order of the Committee of Arrangements,

N. S. DAVIS, Chairman.

